# Multivoxel Pattern Analysis for fMRI Data: A Review

**DOI:** 10.1155/2012/961257

**Published:** 2012-12-06

**Authors:** Abdelhak Mahmoudi, Sylvain Takerkart, Fakhita Regragui, Driss Boussaoud, Andrea Brovelli

**Affiliations:** ^1^Laboratoire d'Informatique, Mathématique, Intelligence Artificielle et Reconnaissance de Formes (LIMIARF), Faculté des Sciences, Université Mohammed V-Agdal, 4 Avenue Ibn Battouta, BP 1014, Rabat, Morocco; ^2^Institut de Neurosciences de la Timone (INT), UMR 7289 CNRS, and Aix Marseille Université, 27 boulevard Jean Moulin, 13385 Marseille, France; ^3^Institut de Neurosciences des Systèmes (INS), UMR 1106 INSERM, and Faculté de Médecine, Aix Marseille Université, 27 boulevard Jean Moulin, 13005 Marseille, France

## Abstract

Functional magnetic resonance imaging (fMRI) exploits blood-oxygen-level-dependent (BOLD) contrasts to map neural activity associated with a variety of brain functions including sensory processing, motor control, and cognitive and emotional functions. The general linear model (GLM) approach is used to reveal task-related brain areas by searching for linear correlations between the fMRI time course and a reference model. One of the limitations of the GLM approach is the assumption that the covariance across neighbouring voxels is not informative about the cognitive function under examination. Multivoxel pattern analysis (MVPA) represents a promising technique that is currently exploited to investigate the information contained in distributed patterns of neural activity to infer the functional role of brain areas and networks. MVPA is considered as a supervised classification problem where a classifier attempts to capture the relationships between spatial pattern of fMRI activity and experimental conditions. In this paper , we review MVPA and describe the mathematical basis of the classification algorithms used for decoding fMRI signals, such as support vector machines (SVMs). In addition, we describe the workflow of processing steps required for MVPA such as feature selection, dimensionality reduction, cross-validation, and classifier performance estimation based on receiver operating characteristic (ROC) curves.

## 1. Classical Statistical Inference in fMRI Research

Functional magnetic resonance imaging (fMRI) exploits blood-oxygen-level-dependent (BOLD) contrasts to map neural activity associated with a variety of brain functions including sensory processing, motor control, and cognitive and emotional functions [1, 2]. BOLD signal changes are due to hemodynamic and metabolic modulations associated with neural activity. BOLD responses mainly reflect synaptic inputs driving neuronal assemblies, rather than their output firing activity [3]. A typical fMRI database contains BOLD signal time courses recorded at multiple voxels in the brain. A voxel is a three-dimensional rectangular cuboid, whose dimensions are in the range of millimeters. In order to map the cerebral areas involved in a given cognitive function, the BOLD signal at each voxel is analysed [4]. Statistical inference is commonly performed using the general linear model (GLM) approach to reveal task-related (or “activated”) brain areas by searching for linear correlations between the fMRI time course and a reference model defined by the experimenter [5–9]. Statistical analysis is then performed iteratively on all voxels to identify brain regions whose BOLD responses display significant statistical effects. This approach is often referred to as mass-univariate model-based analysis, and it represents the gold standard in fMRI research. This approach, however, suffers from several limitations. One of the most compelling things is the assumption that the covariance across neighbouring voxels is not informative about the cognitive function under examination. We will review the statistical methods used in GLM analysis and then present how multivariate and model-free statistical tools based on machine-learning methods overcome these limitations and provide a novel approach in neuroimaging research.

### 1.1. The GLM Approach: Mass Univariate and Model-Based Analysis of fMRI Data

The GLM is normally expressed in matrix formulation by

(1) $$\begin{array}{c} Y=X\beta +\epsilon , \end{array}$$

where *Y* = [*y*_1_,…, *y*_*J*_]^*T*^ is the dependent variable and is a column vector containing the BOLD signal at a single voxel; *ϵ* = [*ϵ*_1_,…, *ϵ*_*J*_]^*T*^ is the error vector whose elements are independent and identically distributed normal random variables with zero mean and variance *σ* ^2^, *ϵ* ~ *N*(0, *σ* ^2^ *I*). *β* = [*β*_1_,…, *β*_*P*_]^*T*^ is the column vector of model parameters where *P* is the number of model parameters; *X* is *J* × *P* design matrix which is a near-complete description of the model. It contains explanatory variables (one row per time point and one column per explanatory variable) quantifying the experimental knowledge about the expected signal.

The parameter estimates of the model that we denote as $\hat{\beta }$ are obtained by minimizing the squared differences between *Y* and the estimated signal $\hat{Y}=X\hat{\beta }$ giving residual errors $\epsilon =Y-\hat{Y}$. The residual sum of squares *S* = ∑_*j*=1_^*J*^ *ϵ*_*j*_^2^ = *ϵ* ^*T*^ *ϵ* is the sum of squared differences between the actual and fitted values and thus measures the fit of the model with these parameter estimates. The least square estimates are the $\hat{\beta }$-values which minimize *S*. This is obtained when

(2) $$\begin{array}{c} \hat{\beta }={\left(X^{T}X\right)}^{-1}X^{T}Y. \end{array}$$

In order to compare experimental conditions, *T*- or *F*-statistics allow to test for a linear combination of $\hat{\beta }$-values that correspond to null hypotheses [10]. For example, to test whether activation in condition *A* is significantly different from activation in condition *B*, a two-sample *t*-test can be used. In this case, the null hypothesis would state that the $\hat{\beta }$-values of the two conditions would not differ, that is, *H*_0_: ${\hat{\beta }}_{A}={\hat{\beta }}_{B}$ or *H*_0_: $(+1){\hat{\beta }}_{A}+(-1){\hat{\beta }}_{B}=0$.

To generalize this argument, we consider linear functions of the beta estimates:

(3) $$\begin{array}{c} c_{1}{\hat{\beta }}_{1}+c_{2}{\hat{\beta }}_{2}+\cdots +c_{P}{\hat{\beta }}_{P}=c^{T}\hat{\beta ,} \end{array}$$

where the constants *c*_*i*_ are the coefficients of a function that “contrasts” the beta estimates ${\hat{\beta }}_{i}$. The vector *c* ^*T*^ = [*c*_1_,…, *c*_*P*_] is referred to as the *contrast* vector. With this definition, *H*_0_ can be then written using a scalar product $c^{T}\hat{\beta }=0$.

To test whether the condition combinations specified in *c* differ significantly from the null hypothesis *H*_0_, the *T*-statistic is computed at each voxel as

(4) $$\begin{array}{c} t=\frac{c^{T}\hat{\beta }}{\sqrt{\text{var}\left(\epsilon \right)c^{T}{\left(X^{T}X\right)}^{-1}c}}. \end{array}$$

**Figure 1:**
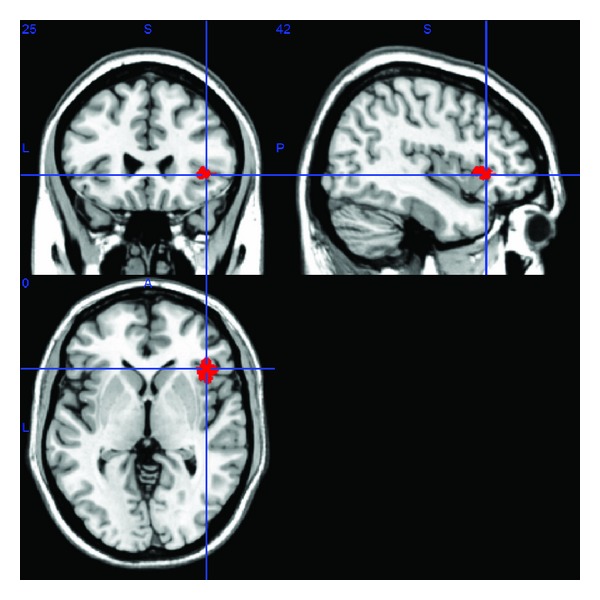
Thresholded statistical map overlaid on anatomical image.

Classical statistical methods, such as *F*-test or ANOVA (analyse of variance), are special cases of the GLM analysis and can be used to perform statistical inference at each voxel. The resulting statistical parametric map (SPM) arises from multiple hypothesis testing (i.e., at all voxels). Classically, the significance level is controlled for family-wise errors using appropriate multiple comparison procedures (e.g., Bonferroni correction). Additionally, Gaussian random field theory (RFT) [11] is used to take into account the spatial smoothness of the statistical map. Instead of assigning a *P* value to each voxel, clusters of voxels are created on the basis of an initial threshold, and then each cluster is assigned a *P* value [5, 12]. The resulting thresholded statistical maps display the brain regions whose BOLD activity significantly correlates with the cognitive functions under investigation (Figure 1).

### 1.2. The Quest for Multivariate and Model-Free fMRI Data Analysis

One of the limitations of the GLM mass-univariate approach is the assumption that the covariance across neighbouring voxels is not informative about the cognitive function under examination. Such covariance is considered as uncorrelated noise and normally reduced using spatial filters that smooth BOLD signals across neighbouring voxels. Additionally, the GLM approach is inevitably limited by the model used for statistical inference.

Multivariate and model-free fMRI methods represent promising techniques to overcome these limitations by investigating the functional role of distributed patterns of neural activity without assuming a specific model. Multivariate model-free methods are based on machine learning and pattern recognition algorithms. Nowadays, multivoxel pattern analysis (MVPA) has become a leading technique in the analysis of neuroimaging data, and it has been extensively used to identify the neural substrates of cognitive functions ranging from visual perception to memory processing [13–16].

The aim of the current paper is to review the mathematical formalism underlying MVPA of fMRI data within the framework of supervised classification tools. We will review the statistical tools currently used and outline the steps required to perform multivariate analysis.

## 2. Multivoxel fMRI Analysis as a Supervised Classification Problem

Multi-voxel pattern analysis (MVPA) involves searching for highly reproducible spatial patterns of activity that differentiate across experimental conditions. MVPA is therefore considered as a supervised classification problem where a classifier attempts to capture the relationships between spatial patterns of fMRI activity and experimental conditions [17].

More generally, classification consists in determining a decision function *f* that takes the values of various “features" in a data “example” *x* and predicts the class of that “example.” “Features” is a generic term used in machine learning to be the set of variables or attributes describing a certain “example.” In the context of fMRI, an “example” may represent a given trial in the experimental run, and the “features” may represent the corresponding fMRI signals in a cluster of voxels. The experimental conditions may represent the different classes.

To obtain the decision function *f*, data (i.e., examples and the corresponding class labels) must be split into two sets: “training set” and “test set.” The classifier is trained using the training set. Training consists of modeling the relationship between the features and the class label by assigning a weight *w* to each feature. This weight corresponds to the relative contribution of the feature to successfully classify two or more classes. When more than two classes are present in the experimental design, the analysis can be transformed into a combination of multiple two-class problems (i.e., each class versus all the others). The classifier is then evaluated with the test set to determine its performance in capturing the relationship between features and classes. Given that there are several data split possibilities (see Section 4), one can train and test many classifiers and end up with one of maximum performance.

Support vector machines (SVMs) [18, 19] have recently become popular as supervised classifiers of fMRI data due to their high performance, their ability to deal with large high-dimensional datasets, and their flexibility in modeling diverse sources of data [20–22]. Furthermore, standard libraries implementing SVMs are available such as SVM-light [23], LIBSVM [24], and PyMVPA [25]. We will therefore review the mathematical basis of SVMs.

### 2.1. Mathematical Basis of Support Vector Machines

#### 2.1.1. Linear SVM

**Figure 2:**
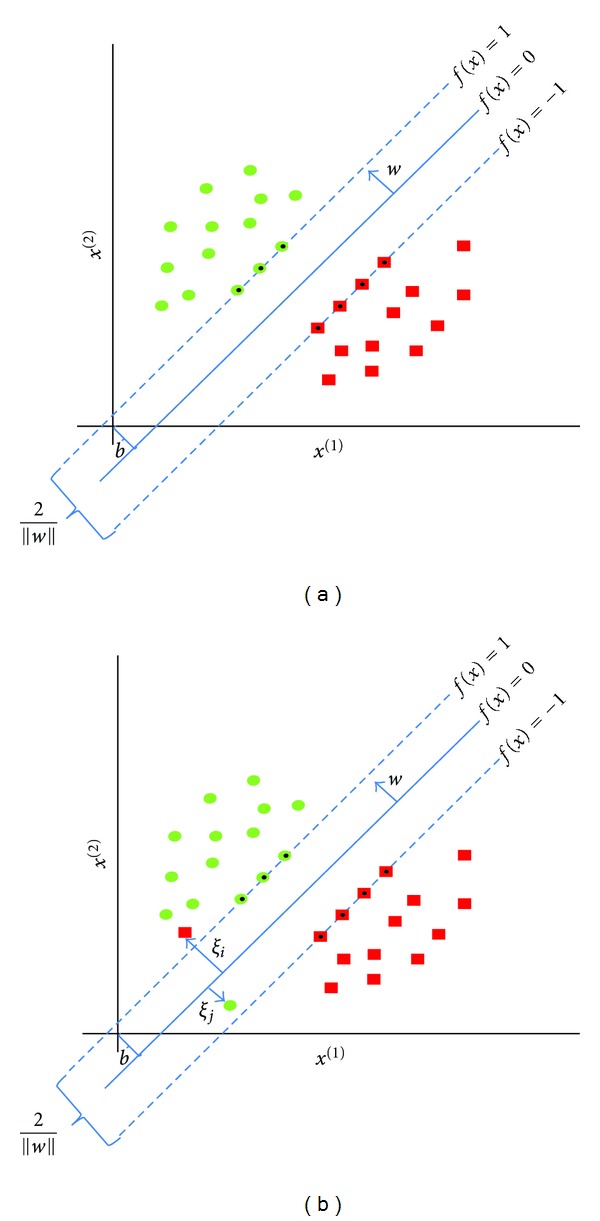
2D space illustration of the decision boundary of the support vector machine (SVM) linear classifier. (a) the hard margin on linearly separable examples where no training errors are permitted. (b) the soft margin where two training errors are introduced to make data nonlinearly separable. Dotted examples are called the support vectors (they determine the margin by which the two classes are separated).

In the simplest linear form of SVMs for two classes, the goal is to estimate a decision boundary (a hyperplane) that separates with maximum margin a set of positive examples from a set of negative examples (Figure 2). Each example is an input vector *x*_*i*_ (*i* = 1,…, *N*) having *M* features (i.e., *x*_*i*_ in *R* ^*M*^) and is associated with one of two classes *y*_*i*_ = −1 or +1. For example, in fMRI research, the data vectors *x*_*i*_ contain BOLD values at discrete time points (or averages of time points) during the experiment, and features could be a set of voxels extracted in each time point; *y* = −1 indicates condition *A*, and *y* = +1 indicates condition B.

If we assume that data are linearly separable, meaning that we can draw a line on a graph of the feature *x* ^(1)^ versus the feature *x* ^(2)^ separating the two classes when *M* = 2 and a hyperplane on graphs of *x* ^(1)^, *x* ^(2)^,…, *x* ^(*M*)^ when *M* > 2, the SVM produces the discriminant function *f* with the largest possible margin:

(5) $$\begin{array}{c} f\left(x\right)=w\cdot x+b. \end{array}$$

*w* is the normal weight vector of the separating hyperplane, *b* is referred to as the “bias,” and it translates the hyperplane away from the origin of the feature space, and · is the inner product:

(6) $$\begin{array}{c} w\cdot x=\sum_{j=1}^{M}w^{\left(j\right)}x^{\left(j\right)}. \end{array}$$

SVM attempts to find the optimal hyperplane *w* · *x* + *b* = 0 which maximizes the margin magnitude 2/||*w*||, that is, it finds *w* and *b* by solving the following *primal* optimization problem:

(7)   min⁡w,b  12||w||2subject  to  yi(xi·w+b)≥1, ∀i∈{1,…,N}.

However, in practice, data are not often linearly separable. To permit training errors and then increase classifier performance, slack variables *ξ*_*i*_ ≥ 0, for  all  *i* ∈ {1,…, *N*}, are introduced:

(8) $$\begin{array}{c} y_{i}\left(x_{i}\cdot w+b\right)\geq 1-\xi_{i},\;\text{for}\;\text{all}\;i\in \left\{1,\ldots ,N\right\},\;\;\xi_{i}\geq 0. \end{array}$$

When *ξ*_*i*_ = 0, for  all  *i* ∈ {1,…, *N*}, (i.e., (7)), the margin is the width of the gap between the classes allowing no training errors, and it is referred to as the “hard margin.” 0 ≤ *ξ*_*i*_ ≤ 1 means that the corresponding training examples are allowed to be inside the gap defined by the hyperplane and the margin. *ξ*_*i*_ ≥ 1 allows some training examples to be misclassified. In such a case, the margin is referred to as “soft margin” (Figure 2).

To control the trade-off between the hyperplane complexity and training errors, a penalty factor *C* is introduced. The *primal* optimization problem becomes

(9)   min⁡w,b,ξ    12||w||2+C∑i=1Nξisubject  to   yi(xi·w+b)−1+ξi≥0,   ∀i∈{1,…,N}, ξi≥0.

**Figure 3:**
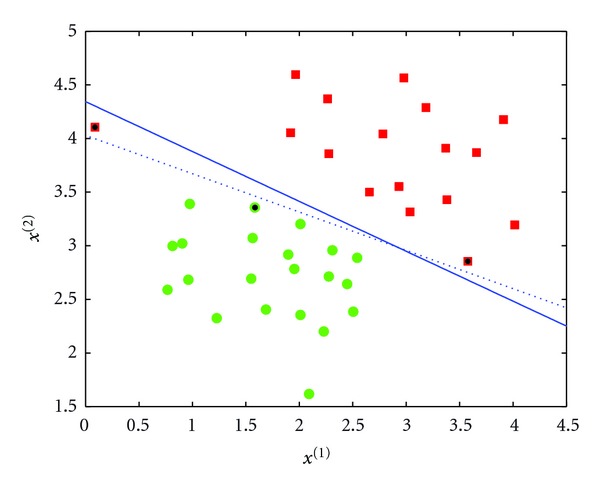
Effect of *C* on the decision boundary. The solid line (*C* = 0.1) allows some training errors (red example on the top left is misclassified). The dashed line (*C* = 10000) does not allow any training error. Even though the *C* = 0.1 case has one misclassification, it represents a trade-off between acceptable classifier performance and overfitting.

High *C* values force slack variables *ξ*_*i*_ to be smaller, approximating the behaviour of hard margin SVM (*ξ*_*i*_ = 0).  Figure 3 shows the effect of *C* on the decision boundary. Large *C*  (*C* = 10000) does not allow any training error. Small *C* (*C* = 0.1) however allows some training errors. In this figure, *C* = 0.1 is typically preferred because it represents a trade-off between acceptable classifier performance and generalization to unseen examples (i.e., overfitting).

To solve the mentioned *primal* optimization problem where a function has to be minimized subject to fixed outside constraints, the method of Lagrange multipliers is used. This method provides a strategy for finding the local maxima and minima of a function subject to equality constraints. These are included in the minimization objective, and Lagrange multipliers allow to quantify how much to emphasize these (see, e.g., [26] for more details).

Let *μ*_*i*_ ≥ 0 and *α*_*i*_ ≥ 0 be two Lagrange multipliers. We derive the so-called *dual* problem using the following Lagrangian *L* of the *primal* problem:

(10) L(w,b,α,ξ,μ)=12||w||2+C∑i=1Nξi−∑i=1Nαi(yi(xiw+b)−1+ξi)−∑i=1Nμiξi.

The Lagrangian *L*(*w*, *b*, *α*, *ξ*, *μ*) needs to be minimized with respect to *w*, *b*, and *ξ* under the constraints *ξ*_*i*_ ≥ 0, *α*_*i*_ ≥ 0, and *μ*_*i*_ ≥ 0  for  all  *i* ∈ {1,…, *N*}. Consequently, the derivatives of *L* with respect to these variables must vanish:

(11) $$\begin{array}{c} \frac{\partial L}{\partial w}=w-\sum_{i=1}^{N}\alpha_{i}y_{i}x_{i}=0, \end{array}$$

(12) $$\begin{array}{c} \frac{\partial L}{\partial b}=\sum_{i=1}^{N}\alpha_{i}y_{i}=0, \end{array}$$

(13) $$\begin{array}{c} \frac{\partial L}{\partial \xi }=C-\alpha_{i}-\mu_{i}=0. \end{array}$$

Substituting the above results in the Lagrange form, we get the following:

(14) $$\begin{array}{c} L\left(w,b,\alpha \right)=\sum_{i=1}^{N}\alpha_{i}-\frac{1}{2}\sum_{i=1}^{N}\sum_{j=1}^{N}\alpha_{i}\alpha_{j}y_{i}y_{j}x_{i}x_{j}. \end{array}$$

According to Lagrange theory, in order to obtain the optimum, it is enough to maximize *L* with respect to *α*_*i*_, for  all  *i* ∈ {1,…, *N*}:

(15)     max
  ∑i=1Nαi−12∑i=1N∑j=1Nαiαjyiyjxixjsubject  to      ∑i=1Nαiyi=0,   ∀i∈{1,…,N} 0≤αi≤C,

where *α*_*i*_ ≤ *C* comes from *μ*_*i*_ ≥ 0 and *C* − *α*_*i*_ − *μ*_*i*_ = 0.

Because this *dual* problem has a quadratic form, the solution can be found iteratively by quadratic programming (QP), sequential minimal optimization (SMO), or least square (LS). This solution has the property that *w* is a linear combination of a few of the training examples:

(16) $$\begin{array}{c} w=\sum_{i=1}^{N}\alpha_{i}y_{i}x_{i},\;\forall \alpha_{i}\geq 0. \end{array}$$

The key feature of this equation is that *α*_*i*_ = 0 for every *x*_*i*_ except those which are inside the margin. Those are called the *support vectors*. They lie closest to the decision boundary and determine the margin. Note that if all *nonsupport vectors* were removed, the same maximum margin hyperplane would be found.

In practice, most fMRI experimenters use linear SVMs because they produce linear boundaries in the original feature space, which makes the interpretation of their results straightforward. Indeed in this case, examining the weight maps directly allows the identification of the most discriminative features [27].

#### 2.1.2. Nonlinear SVM

Nonlinear SVMs are often used for discrimination problems when the data are nonlinearly separable. Vectors are mapped to a high-dimensional feature space using a function *g*(*x*).

In nonlinear SVMs, the decision function will be based on the hyperplane:

(17) $$\begin{array}{c} f\left(x\right)=\sum_{i=1}^{N}\alpha_{i}y_{i}g\left(x_{i}\right)\cdot g\left(x\right)+b. \end{array}$$

A mathematical tool known as “kernel trick” can be applied to this equation which solely depends on the dot product between two vectors. It allows a nonlinear operator to be written as a linear one in a space of higher dimension. In practice, the dot product is replaced by a “kernel function” *k*(*x*, *x*′) = *g*(*x*) · *g*(*x*′) which does not need to be explicitly computed reducing the optimization problem to the linear case:

(18) $$\begin{array}{c} f\left(x\right)=\sum_{i=1}^{N}\alpha_{i}y_{i}k\left(x_{i},x\right)+b. \end{array}$$

Several types of kernels can be used in SVMs models. The most common kernels are polynomial kernels and radial basis functions (RBFs).

The polynomial kernel is defined by

(19) $$\begin{array}{c} k_{d,K}\left(x,x^{\prime }\right)={\left(x\cdot x\prime +K\right)}^{d}. \end{array}$$

**Figure 4:**
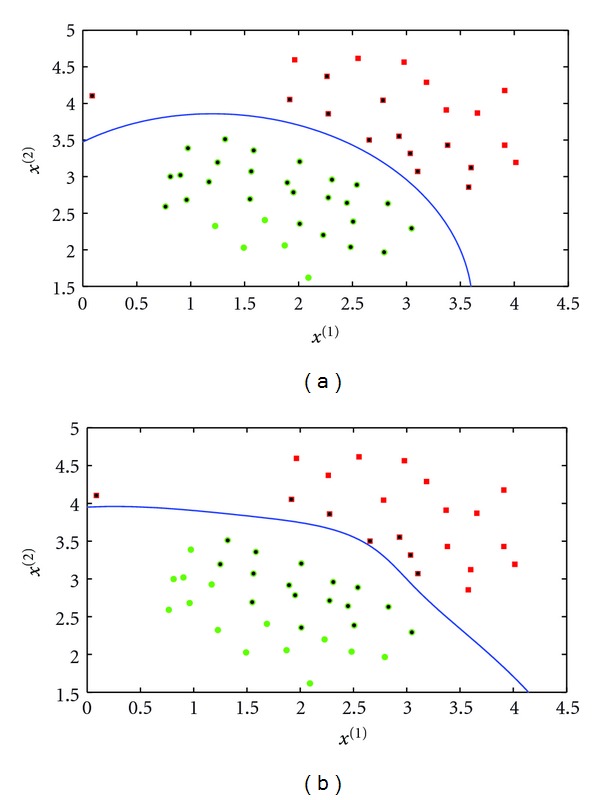
Decision boundary with polynomial kernel. *d* = 2 (a) and *d* = 4 (b). *K* is set to 0.

The *K* and *d* parameters are set to control the decision boundary curvature.  Figure 4 shows the decision boundary with two different values of *d* and *K* = 0. We note that the case with *K* = 0 and *d* = 1 is a linear kernel.

**Figure 5:**
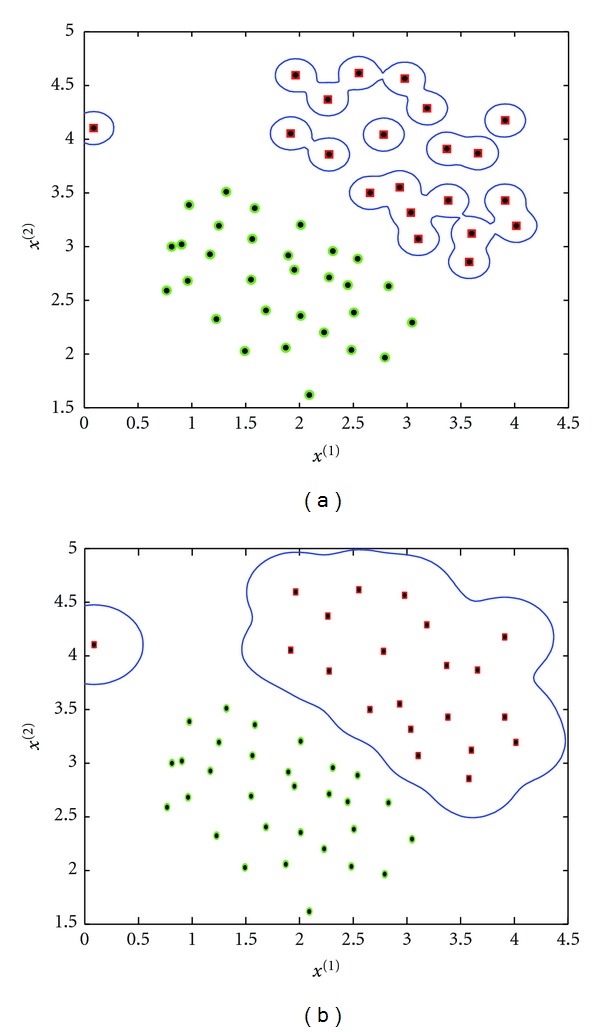
Decision boundary with RBF kernel. *σ* = 0.1 (a) and *σ* = 0.2 (b).

Radial basis function (RBF) kernel is defined by

(20) $$\begin{array}{c} k_{\sigma }\left(x,x^{\prime }\right)=\exp\left(-\frac{{||x-x^{\prime }||}^{2}}{2\sigma^{2}}\right), \end{array}$$

where *σ* is a hyperparameter. A large *σ* value corresponds to a large kernel width. This parameter controls the flexibility of the resulting classifier (Figure 5).

In the fMRI domain, although non-linear transformations sometimes provide higher prediction performance, their use limits the interpretation of the results when the feature weights are transformed back to the input space [28].

### 2.2. Comparison of Classifiers and Preprocessing Strategies

Although SVMs are efficient at dealing with large high-dimensional data-sets, they are, as many other classifiers, affected by preprocessing steps such as spatial smoothing, temporal detrending, and motion correction. LaConte et al. [27] compared SVMs to canonical variate analysis (CVA) and examined their relative sensitivity with respect to ten combinations of pre-processing steps. The study showed that for both SVM and CVA, classification of individual time samples of whole brain data can be performed with no averaging across scans. Ku et al. [29] compared four pattern recognition methods (SVM, Fisher linear discriminant (FLD), correlation analysis (CA), and Gaussian naive bayes (GNB)) and found that classifier performance can be improved through outlier elimination. Misaki et al. [30] compared six classifiers attempting to decode stimuli from response patterns: pattern correlation, k-nearest neighbors (KNN), FLD, GNB, and linear and nonlinear SVM. The results suggest that normalizing mean and standard deviation of the response patterns either across stimuli or across voxels had no significant effect.

On the other hand, classifier performance can be improved by reducing the data dimensionality or by selecting a set of discriminative features. Decoding performance was found to increase by applying dimensionality reduction using the recursive features elimination (RFE) algorithm [31] or after selection of independent voxels with highest overall responsiveness, using a priori knowledge of GLM measures [29]. However, LaConte et al. [27] showed that classification of whole brain data can be performed with no prior feature selection, while Mourão-Miranda et al. [32] found that SVM was more accurate compared to FLD when classifying brain states without prior selection of spatial features. Schmah et al. [33] compared, in terms of performance, a set of classification methods (adaptive FLD, adaptive quadratic discriminant (QD), GNB, linear and nonlinear SVM, logistic regression (LR), restricted Boltzmann machines (RBM), and KNN) applied to the fMRI volumes without reducing dimensionality and showed that the relative performance varied considerably across subjects and classification tasks.

Other studies attempted to compare classifiers in terms of their performances or execution time. Cox and Savoy [14] studied linear discriminant (LD) and SVMs to classify patterns of fMRI activation evoked by the visual presentation of various categories of objects. The classifier accuracy was found to be significant for both linear and polynomial SVMs compared to the LD classifier. Pereira and Botvinick [34] found that the GNB classifier is a reasonable choice for quick mapping, LD is likely preferable if more time is given, and linear SVM can achieve the same level of performance if the classifier parameters are well set using cross-validation (see Section 4).

## 3. Feature Selection and Dimensionality Reduction

When dealing with single-subject univariate analysis, features may be created from the maps estimated using a GLM. A typical feature will consist of the pattern of *β*-values across voxels. The analysis is normally performed on spatially unsmoothed data to preserve fine-grained subject-specific information [35]. In such a case, features are simply the voxels. Other authors recommend applying spatial smoothing [36]. This idea is highly debated in the fMRI literature [30, 37] (see also Section 2.2). In both cases, the feature space can still be considered as high dimensional when all brain voxels (or at least too-large regions of interest) are used. Therefore, the dimensionality of the data needs to be significantly reduced, and informative features (voxels) have to be wisely selected in order to make the classification task feasible. When small regions of interest are used, there is typically no need to reduce the dimensionality (see the following Section 3.1).

Several studies demonstrated the relevance of feature selection. Pearson's and Kendall *τ* rank correlation coefficient have been used to evaluate the elements of the functional connectivity matrix between each pair of brain regions as classification features [38], whereas voxel reliability and mutual information metrics have been compared for identifying subsets of voxels in the fMRI data which optimally distinguish object identity [39]. Åberg and Wessberg [40] explored the effectiveness of evolutionary algorithms in determining a limited number of voxels that optimally discriminate between single volumes of fMRI. The method is based on a simple multiple linear regression classifier in conjunction with as few as five selected voxels which outperforms the feature selection based on statistical parametric mapping (SPM) [41].

More recently, novel techniques have been developed to find informative features while ignoring uninformative sources of noise, such as principal components analysis (PCA) and independent component analysis (ICA) [42, 43]. Such methods perform well when dealing with single-subject analysis. Recently, attempts have been made to extend these methods to group-level analysis by developing group ICA approaches to extract independent components from the analysis of subject's group data [44, 45].

It is worth mentioning that feature selection can be improved by the use of cross-validation (see Section 4). The best classifier will generally include only a subset of features that are deemed truly informative. In fact, SVM classifiers can also be used to perform feature selection. To do so, Martino et al. [31] developed the recursive feature elimination (RFE) algorithm which iteratively eliminates the least discriminative features based on multivariate information as detected by the classifier. For each voxel selection level, the RFE consists of two steps. First, an SVM classifier is trained on a subset of training data using the current set of voxels. Second, a set of voxels is discarded according to their discriminative weights as estimated during training. Data used as test are classified, and generalization performance is assessed at each iteration. RFE has been recently used for the analysis of fMRI data and has been proven to improve generalization performances in discriminating visual stimuli during two different tasks [31, 46].

### 3.1. Regions of Interest (ROI): Searchlight Analysis

Multivariate classification methods are used to identify whether the fMRI signals from a given set of voxels contain a dissociable pattern of activity according to experimental manipulation. One option is to analyze the pattern of activity across all brain voxels. In such a case, the number of voxels exceeds the number of training patterns which makes the classification computationally expensive.

A typical approach is to make assumptions about the anatomical regions of interest (ROI) suspected to be correlated with the task [14, 47, 48]. In such cases, the ROI will represent spatially contiguous sets of voxels, but not necessarily adjacent.

**Figure 6:**
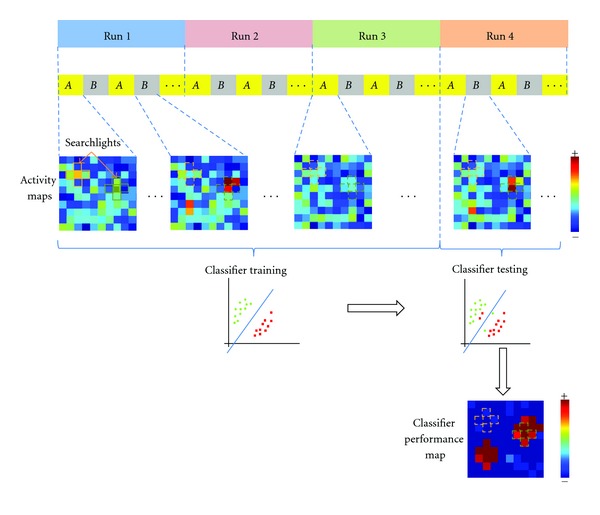
2D illustration of the “searchlight” method on simulated maps of 10 × 10 pixels. For each pixel in the activity map, 5 neighbors (a searchlight) are extracted to form a feature vector. Extracted searchlights from the activity maps of each condition (*A* or *B*) form then the input examples. A classifier is trained using training examples (corresponding to the 3 first runs) and tested using the examples of the fourth run. The procedure is then repeated along the activity maps for each pixel to produce finally a performance map that shows how well the signal in the local neighborhoods differentiates the experimental conditions *A* and *B*.

An alternative is to select fewer voxels (e.g., those within a sphere centred at a voxel) and repeat the analysis at all voxels in the brain. This method has been introduced by Kriegeskorte et al. [49], and it has been named “searchlight.” It produces a multivariate information map where each voxel is assigned the classifier's performance. In other terms, the searchlight method scores a voxel by how accurately the classifier can predict a condition of each example on the training set, based on the data from the voxel and its immediately adjacent neighbours.  Figure 6 shows a 2D illustration of the searchlight method applied to 120 simulated maps of 10 × 10 pixels. The pixels for conditions *A* are random numbers, and pixels of condition *B* are constructed from those of *A* except in some patterns where a value of 1 is added. We used four runs where each run contains 30 examples (15 for condition *A* and 15 for condition *B*).

**Figure 7:**
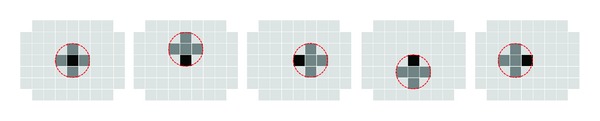
Illustration of the Monte Carlo fMRI brain mapping method in one voxel (in black). Instead of centering the search volume (dashed-line circle) at the voxel as in the searchlight method and computing a single performance for it, here the voxel is included in five different constellations with other neighboring voxels (dark gray). In each constellation, a classification performance is computed for it. In the end, the average performance across all the constellations is assigned to the dark voxel.

More recently, Björnsdotter et al. [50] proposed a Monte Carlo approximation of the searchlight designed for fast whole brain mapping. One iteration of the algorithm consists of the brain volume being randomly divided into a number of clusters (search spheres) such that each voxel is included in one (and only one) cluster, and a classifier performance is computed for it. Thus, a mean performance across all the constellations in which the voxel took part is assigned to that voxel (as opposed to the searchlight where each voxel is assigned the one value computed when the sphere was centered on it) (Figure 7).

## 4. Performance Estimation and Cross-Validation

**Figure 8:**
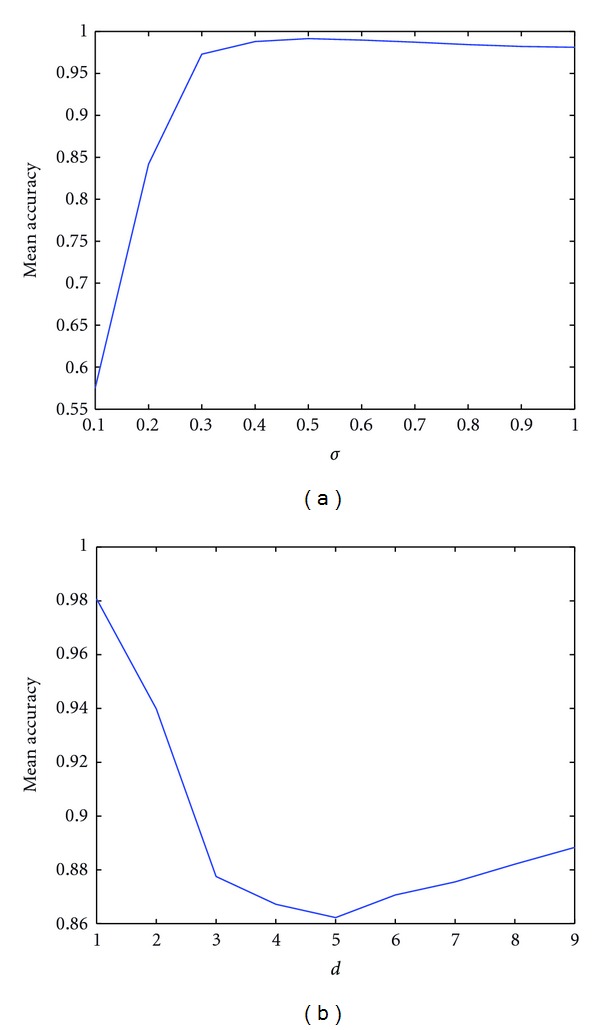
Mean accuracy after 4-fold cross-validation to classify the data shown in Figure 3. The parameters showing the best accuracy are *d* = 1 for polynomial kernel and *σ* ≥ .4 for RBF kernel.

To ensure unbiased testing, the data must be split into two sets: a training and test set. In addition: it is generally recommended to choose a larger training set in order to enhance classifier convergence. Indeed, the performance of the learned classifier depends on how the original data are partitioned into training and test set, and, most critically, on their size. In other words, the more instances we leave for test, the fewer samples remain for training, and hence the less accurate becomes the classifier. On the other hand, a classifier that explains one set of data well does not necessarily generalize to other sets of data even if the data are drawn from the same distribution. In fact, an excessively complex classifier will tend to overfit (i.e., it will fail to generalize to unseen examples). This may occur, for example, when the number of features is too large with respect to the number of examples (i.e., *M* ≫ *N*). This problematic is known as “the curse of dimensionality” [51]. One way to overcome this problem is the use of “cross-validation.” This procedure allows efficient evaluation of the classifier performance [52–54]. The goal is to identify the best parameters for the classifier (e.g., parameters *C*, *d*, and *σ*) that can accurately predict unknown data (Figure 8). By cross-validation, the same dataset can be used for both the training and testing of the classifier, thus increasing the number of examples *N* with the same number of features *M*.

### 4.1. N-Fold Cross-Validation

**Figure 9:**
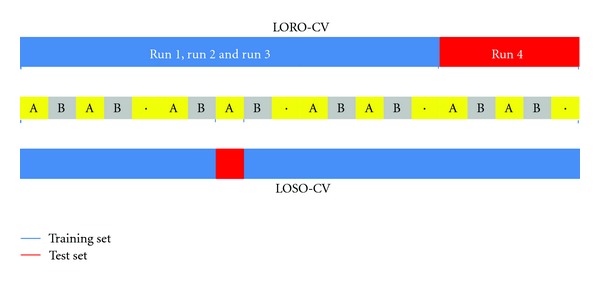
Leave-one-run-out cross-validation (LORO-CV) and leave-one-sample-out cross-validation (LOSO-CV). A classifier is trained using training set (in blue) and then tested using the test set (in red) to get a performance. This procedure is repeated for each run in LORO-CV and for each sample in LOSO-CV to get at the end an averaged performance.

In *N*-fold cross-validation, the original data are randomly partitioned into *N* subsamples. Of the *N* subsamples, a single subsample is retained for validating the model, and the remaining *N* − 1 subsamples are used as training data. The cross-validation procedure is then repeated *N* times, each of the *N* sub-samples being used for testing. The *N* results can be averaged (or otherwise combined) to produce single performance estimation. Two schemes of cross-validation are used for single-subject MVPA (Figure 9). The first one is the leave-one-run-out cross-validation (LORO-CV). In this procedure, data of one run provide the test samples, and the remaining runs provide the training samples. The second one is leave-one-sample-out cross-validation (LOSO-CV) in which one sample is taken from each class as a test sample, and all remaining samples are used for classifier training. The samples are randomly selected such that each sample appears in the test set at least once. LOSO-CV produces higher performances than the LORO-CV but is computationally more expensive due to a larger number of training processes [30].

### 4.2. Classifier Performance

**Figure 10:**
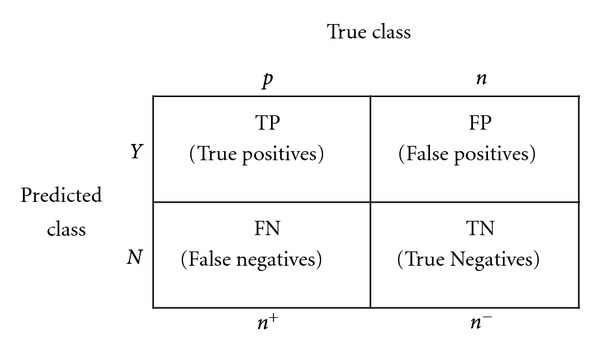
*Confusion* matrix for performance evaluation.

Machine-learning algorithms come with several parameters that can modify their behaviors and performances. Evaluation of a learned model is traditionally performed by maximizing an accuracy metric. Considering a basic two-class classification problem, let {*p*, *n*} be the true positive and negative class labels, and let {*Y*, *N*} be the predicted positive and negative class labels. Then, a representation of classification performance can be formulated by a *confusion* matrix (contingency table), as illustrated in Figure 10. Given a classifier and an example, there are four possible outcomes. If the example is positive and it is classified as positive, it is counted as a true positive (TP); if it is classified as negative, it is counted as a false negative (FN). If the example is negative and it is classified as negative, it is counted as a true negative (TN); if it is classified as positive, it is counted as a false positive (FP). Following this convention, the accuracy metric is defined as

(21) $$\begin{array}{c} \text{ACC}=\frac{\text{TP}+\text{TN}}{n^{+}+n^{-}}, \end{array}$$

where *n* ^+^ and *n* ^−^ are the number of positive and negative examples, respectively (*n* ^+^ = TP + FN, *n* ^−^ = FP + TN). However, accuracy can be deceiving in certain situations and is highly sensitive to changes in data. In other words, in the presence of *unbalanced* data-sets (i.e., where *n* ^+^ ≫ *n* ^−^), it becomes difficult to make relative analysis when the evaluation metric is sensitive to data distributions. In fMRI tasks, the experimental designs are often *balanced* (same fraction of conditions of each type in each run), but there are cases where they are *unbalanced*. Furthermore, any use of random cross-validation procedure to evaluate a classifier may cause data-sets to *unbalance*.

#### 4.2.1. Receiver Operating Characteristic (ROC) Curve

Metrics extracted from the receiver operating characteristic (ROC) curve can be a good alternative for model evaluation, because they allow the dissociation of errors on positive or negative examples. The ROC curve is formed by plotting true positive rate (TPR) over false positive rate (FPR) defined both from the *confusion* matrix by

(22) $$\begin{array}{c} \text{TPR}=\frac{\text{TP}}{n^{+}},\\ \text{FPR}=\frac{\text{FP}}{n^{-}}. \end{array}$$

Any point (FPR; TPR) in ROC space corresponds to the performance of a single classifier on a given distribution. The ROC space is useful because it provides a visual representation of the relative trade-offs between the benefits (reflected by TP) and costs (reflected by FP) of classification in regards to data distributions.

Generally, the classifier's output is a continuous numeric value. The decision rule is performed by selecting a decision threshold which separates the positive and negative classes. Most of the time, this threshold is set regardless of the class distribution of the data. However, given that the optimal threshold for a class distribution may vary over a large range of values, a pair (FPR; TPR) is thus obtained at each threshold value. Hence, by varying this threshold value, an ROC curve is produced.

**Figure 11:**
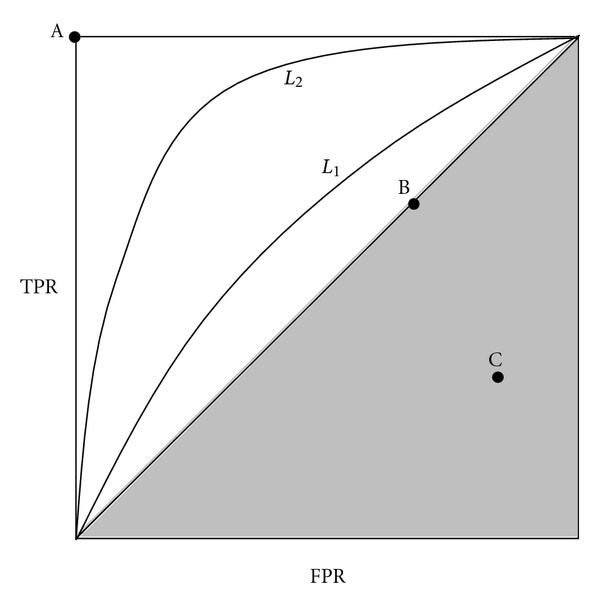
ROC curve representation.

Figure 11 illustrates a typical ROC graph with points A, B, and C representing ROC points and curves *L*_1_ and *L*_2_ representing ROC curves. According to the structure of the ROC graph, point A (0,1) represents a perfect classification. Generally speaking, one classifier is better than another if its corresponding point in ROC space is closer to the upper left hand corner. Any classifier whose corresponding ROC point is located on the diagonal, such as point B, is representative of a classifier that will provide a random guess of the class labels (i.e., a random classifier). Therefore, any classifier that appears in the lower right triangle of ROC space performs worse than random guessing, such as the classifier associated with point C in the shaded area.

In order to assess different classifier's performances, one generally uses the area under the ROC curve (AUC) as an evaluation criterion [55]. For instance, in Figure 11, the *L*_2_ curve provides a larger AUC measure compared to that of *L*_1_; therefore, the corresponding classifier associated with *L*_2_ provides better performance compared to the classifier associated with *L*_1_. The AUC has an important statistical property: it is equivalent to the probability that the classifier will evaluate a randomly chosen positive example higher than a randomly chosen negative example. Smith and Nichols [56] have shown that the AUC is a better measure of classifier performance than the accuracy measure.

Processing the AUC would need the computation of an integral in the continuous case; however, in the discrete case, the area is given by [57]

(23) $$\begin{array}{c} \text{AUC}=\frac{\sum_{i=1}^{n^{-}}\sum_{j=1}^{n^{-}}1_{f(x^{+})>f(x^{-})}}{n^{+}n^{-}}, \end{array}$$

where *f* is the decision function of the discrete classifier, *x* ^+^ and *x* ^−^, respectively, denote the positive and negative examples, and 1_*π*_ is defined to be 1 if the predicate *π* holds and 0 otherwise. This equation states that if a classifier *f*(*x*) is such that *f*(*x*_*i*_^+^) > *f*(*x*_*j*_^−^), for  all  *i* = 1,…, *n* ^+^, for  all  *j* = 1,…, *n* ^−^, then the AUC of this classifier is maximal. Any negative example that happens to be ranked higher than positive examples makes the AUC decreases.

#### 4.2.2. An Example of ROC Curve Applied to SVM Classifier

SVMs can be used as classifiers that output a continuous numeric value in order to plot the ROC curve. In fact, in standard SVM implementations, the continuous output *f* of a test example *x* (i.e., *f* = *w* · *x* + *b*) is generally fed into a sign function: if sign⁡(*f*) = +1, the example *x* is considered as positive and inversely if sign⁡(*f*) = −1, *x* is considered as negative (as if a threshold *t* is frozen at *t* = 0 and *x* is positive if *f* > *t*, and is negative if *f* < *t*). In this case, a single pair of FPR; TPR is obtained. Thus, if one could vary the threshold *t* in a range between the maximum and minimum of all the outputs *f* of the test set (min(*f*) ≤*t*≤ max(*f*)), the ROC curve could be obtained. The algorithm will thus follow the following steps.

- Step  1. Compute the output vector *f* for all examples in the test set.
- Step  2. For each value of a threshold *t* between minimum and maximum of *f*,
  - Step 2.1. compute sign⁡(*f* + *t*) and assign examples to the corresponding classes;
  - Step 2.2. plot the corresponding point (FPR; TPR).

**Figure 12:**
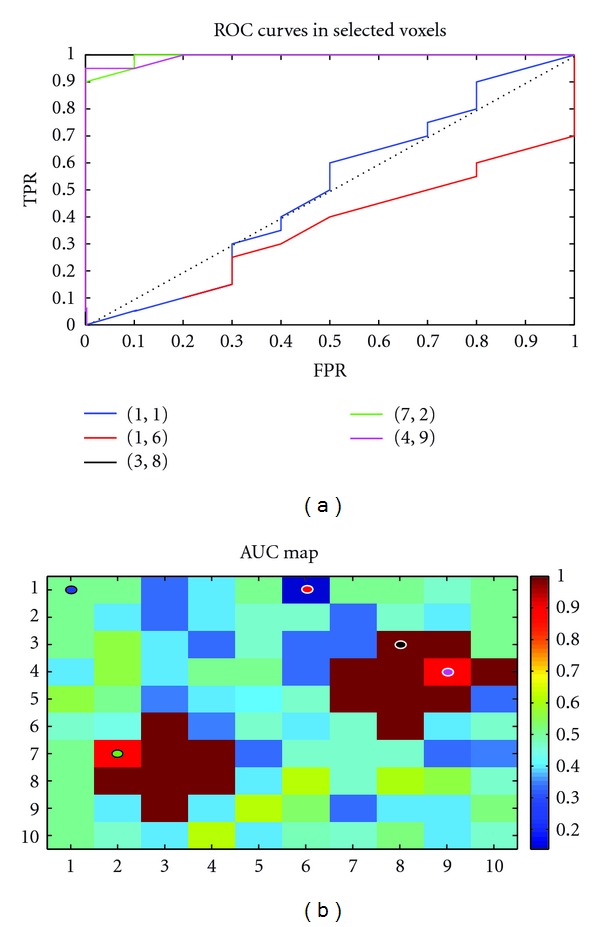
ROC analysis of *unbalanced* simulated data. Data in Figure 6 were *unbalanced* in order to show the threshold effect. (a) ROC curves corresponding to some coordinates (voxels) shown in colored circles in the AUC map in (b).

We performed this procedure on the simulated data used for the searchlight analysis. However, data were unbalanced in order to show the threshold effect (we used four runs each containing 30 exampls, 10 for condition *A* and 20 for condition *B*).  Figure 12 shows the ROC curves corresponding to different voxels. The area under the ROC curve is computed for all voxels yielding the AUC map in Figure 12.

A last point worth mentioning is that the classifier performance measures its ability to generalize to unseen data under the assumption that training and test examples are drawn from the same distribution. However, this assumption could be violated when using cross-validation [34]. An alternative could be the use of Bayesian strategies for model selection given their efficiency both in terms of computational complexity and in terms of the available degrees of freedom [58].

#### 4.2.3. Nonparametric Permutation Test Analysis

Nonparametric permutation test analysis was introduced in functional neuroimaging studies to provide flexible and intuitive methodology to verify the validity of the classification results [59, 60]. The significance of a statistic expressing the experimental effect can be assessed by comparison with the distribution of values obtained when the labels are permuted [61].

Concretely, to verify the hypothesis *H*_0_ under which there is no difference between conditions *A* and *B* when the class labels are randomly permuted, one can follow these steps: (1) permute the labels on the sample; (2) compute the maximum *t-*statistic; (3) repeat over many permutations; (4) obtain a distribution of values for the *t-*statistic; (5) find the threshold corresponding to a given *P* value determining the degree of rejection of the hypothesis [62, 63].

In particular experimental conditions when the fMRI data exhibit temporal autocorrelation [64], an assumption of “exchangeability” of scans (i.e., rearranging the labels on the scans without affecting the underlying distribution of possible outcomes) within subjects is not tenable. In this case, to analyze a group of subjects for population inference, one exclusively assumes exchangeability of subjects. Nichols and Holmes [60] presented practical examples from functional neuroimaging both in single-subject and multisubject experiments, and Golland and Fischl. [62] proposed practical recommendations on performing permutation tests for classification.

## 5. Conclusion

In this paper, we have reviewed how machine-learning classifier analysis can be applied to the analysis of functional neuroimaging data. We reported the limitations of univariate model-based analysis and presented the multivariate model-free analysis as a solution. By reviewing the literature comparing different classifiers, we focused on support vector machine (SVM) as supervised classifier that can be considered as an efficient tool to perform multivariate pattern analysis (MVPA). We reported the importance of feature selection and dimensionality reduction for the success of the chosen classifier in terms of performance, and the importance of a cross-validation scheme both in selecting the best parameters for the classifier and computing the performance. The use of ROC curves seems to be more accurate to evaluate the classifier performance, while nonparametric permutation tests provide flexible and intuitive methodology to verify the validity of the classification results.

## References

[1] Ogawa S, Lee TM, Kay AR, Tank DW (1990). Brain magnetic resonance imaging with contrast dependent on blood oxygenation. *Proceedings of the National Academy of Sciences of the United States of America*.

[2] Kwong KK, Belliveau JW, Chesler DA (1992). Dynamic magnetic resonance imaging of human brain activity during primary sensory stimulation. *Proceedings of the National Academy of Sciences of the United States of America*.

[3] Logothetis NK, Pauls J, Augath M, Trinath T, Oeltermann A (2001). Neurophysiological investigation of the basis of the fMRI signal. *Nature*.

[4] Jezzard P, Matthews MP, Smith MS (2003). Functional MRI: an introduction to methods. *Journal of Magnetic Resonance Imaging*.

[5] Friston KJ, Frith CD, Liddle PF, Frackowiak RSJ (1991). Comparing functional (PET) images: the assessment of significant change. *Journal of Cerebral Blood Flow and Metabolism*.

[6] McIntosh AR, Grady CL, Haxby JV, Maisog JM, Horwitz B, Clark CM (1996). Within-subject transformations of PET regional cerebral blood ow data: ANCOVA, ratio, and z-score adjustments on empirical data. *Human Brain Mapping*.

[7] Friston KJ, Holmes AP, Price CJ, Büchel C, Worsley KJ (1999). Multisubject fMRI studies and conjunction analyses. *NeuroImage*.

[8] McKeown MJ, Makeig S, Brown GG (1998). Analysis of fMRI data by blind separation into independent spatial components. *Human Brain Mapping*.

[9] Kjems U, Hansen LK, Anderson J (2002). The quantitative evaluation of functional neuroimaging experiments: mutual information learning curves. *NeuroImage*.

[10] Frackowiak RSJ, Friston KJ, Frith C (2003). *Human Brain Function*.

[11] Brett M, Penny W, Kiebel S (2004). *Introduction to Random Field Theory*.

[12] Cox DR, Miller HD (1965). *The Theory of Stochastic Processes*.

[13] Norman KA, Polyn SM, Detre GJ, Haxby JV (2006). Beyond mind-reading: multi-voxel pattern analysis of fMRI data. *Trends in Cognitive Sciences*.

[14] Cox DD, Savoy RL (2003). Functional magnetic resonance imaging (fMRI) “brain reading”: detecting and classifying distributed patterns of fMRI activity in human visual cortex. *NeuroImage*.

[15] Haxby JV, Gobbini MI, Furey ML, Ishai A, Schouten JL, Pietrini P (2001). Distributed and overlapping representations of faces and objects in ventral temporal cortex. *Science*.

[16] Downing PE, Wiggett AJ, Peelen MV (2007). Functional magnetic resonance imaging investigation of overlapping lateral occipitotemporal activations using multi-voxel pattern analysis. *Journal of Neuroscience*.

[17] Davatzikos C, Ruparel K, Fan Y (2005). Classifying spatial patterns of brain activity with machine learning methods: application to lie detection. *NeuroImage*.

[18] Cortes C, Vapnik V (1995). Support-vector networks. *Machine Learning*.

[19] Vapnik VN (1995). *The Nature of Statistical Learning Theory*.

[20] Timothy M, Alok D, Svyatoslav V (2012). Support Vector Machine classification and characterization of age-related reorganization of functional brain networks. *NeuroImage*.

[21] Formisano E, De Martino F, Valente G (2008). Multivariate analysis of fMRI time series: classification and regression of brain responses using machine learning. *Magnetic Resonance Imaging*.

[22] Hanson SJ, Halchenko YO (2008). Brain reading using full brain Support Vector Machines for object recognition: there is no “face” identification area. *Neural Computation*.

[23] Joachims T (2002). *Learning to Classify Text Using Support Vector Machines*.

[24] Chang CC, Lin CJ (2011). LIBSVM: a library for Support Vector Machines. *ACM Transactions on Intelligent Systems and Technology*.

[25] Hanke M, Halchenko YO, Sederberg PB, Hanson SJ, Haxby JV, Pollmann S (2009). PyMVPA: a python toolbox for multivariate pattern analysis of fMRI data. *Neuroinformatics*.

[26] Boyd S, Vandenberghe L (2004). *Convex Optimization*.

[27] LaConte S, Strother S, Cherkassky V, Anderson J, Hu X (2005). Support Vector Machines for temporal classification of block design fMRI data. *NeuroImage*.

[28] Brodersen KH, Schofield TM, Leff AP (2011). Generative embedding for Model-Based classification of FMRI data. *PLoS Computational Biology*.

[29] Ku SP, Gretton A, Macke J, Logothetis NK (2008). Comparison of pattern recognition methods in classifying high-resolution BOLD signals obtained at high magnetic field in monkeys. *Magnetic Resonance Imaging*.

[30] Misaki M, Kim Y, Bandettini PA, Kriegeskorte N (2010). Comparison of multivariate classifiers and response normalizations for pattern-information fMRI. *NeuroImage*.

[31] De Martino F, Valente G, Staeren N, Ashburner J, Goebel R, Formisano E (2008). Combining multivariate voxel selection and Support Vector Machines for mapping and classification of fMRI spatial patterns. *NeuroImage*.

[32] Mourão-Miranda J, Bokde ALW, Born C, Hampel H, Stetter M (2005). Classifying brain states and determining the discriminating activation patterns: support vector machine on functional MRI data. *NeuroImage*.

[33] Schmah T, Yourganov G, Zemel RS, Hinton GE, Small SL, Strother SC (2010). Comparing classification methods for longitudinal fMRI studies. *Neural Computation*.

[34] Pereira F, Botvinick M (2011). Information mapping with pattern classifiers: a comparative study. *NeuroImage*.

[35] Kamitani Y, Sawahata Y (2010). Spatial smoothing hurts localization but not information: pitfalls for brain mappers. *NeuroImage*.

[36] Op de Beeck HP (2010). Against hyperacuity in brain reading: spatial smoothing does not hurt multivariate fMRI analyses?. *NeuroImage*.

[37] Swisher JD, Gatenby JC, Gore JC (2010). Multiscale pattern analysis of orientation-selective activity in the primary visual cortex. *Journal of Neuroscience*.

[38] Shen H, Wang L, Liu Y, Hu D (2010). Discriminative analysis of resting-state functional connectivity patterns of schizophrenia using low dimensional embedding of fMRI. *NeuroImage*.

[39] Sayres R, Ress D, Spector KG Identifying distributed object representations in human extrastriate visual cortex.

[40] Åberg MB, Wessberg J (2008). An evolutionary approach to the identification of informative voxel clusters for brain state discrimination. *IEEE Journal on Selected Topics in Signal Processing*.

[41] Kiebel SJ, Friston KJ (2004). Statistical parametric mapping for event-related potentials: I. Generic considerations. *NeuroImage*.

[42] Hyvärinen A, Oja E (2000). Independent component analysis: algorithms and applications. *Neural Networks*.

[43] Rowe DB, Hoffmann RG (2006). Multivariate statistical analysis in fMRI. *IEEE Engineering in Medicine and Biology Magazine*.

[44] Schöpf V, Windischberger C, Robinson S (2011). Model-free fMRI group analysis using FENICA. *NeuroImage*.

[45] Rombouts SARB, Damoiseaux JS, Goekoop R (2009). Model-free group analysis shows altered BOLD FMRI networks in dementia. *Human Brain Mapping*.

[46] Chu C, Hsu A-L, Chou K-H, Bandettini P, Lin C (2011). Does feature selection improve classification accuracy? Impact of sample size and feature selection on classification using anatomical magnetic resonance images. *NeuroImage*.

[47] Haynes JD, Rees G (2005). Predicting the orientation of invisible stimuli from activity in human primary visual cortex. *Nature Neuroscience*.

[48] Kamitani Y, Tong F (2005). Decoding the visual and subjective contents of the human brain. *Nature Neuroscience*.

[49] Kriegeskorte N, Goebel R, Bandettini P (2006). Information-based functional brain mapping. *Proceedings of the National Academy of Sciences of the United States of America*.

[50] Björnsdotter M, Rylander K, Wessberg J (2011). A Monte Carlo method for locally multivariate brain mapping. *NeuroImage*.

[51] Bellman RE (1961). *Adaptive Control Processes—A Guided Tour*.

[52] Kohavi R . A study of cross-validation and bootstrap for accuracy estimation and model selection.

[53] Lemm S, Blankertz B, Dickhaus T, Müller KR (2011). Introduction to machine learning for brain imaging. *NeuroImage*.

[54] Hastie T, Tibshirani R, Friedman J (2009). *The Elements of Statistical Learning: Data Mining, Inference, and Prediction*.

[55] Fawcett T (2006). An introduction to ROC analysis. *Pattern Recognition Letters*.

[56] Smith SM, Nichols TE (2009). Threshold-free cluster enhancement: addressing problems of smoothing, threshold dependence and localisation in cluster inference. *NeuroImage*.

[57] Yan L, Dodier R, Mozer MC, Wolniewicz R Optimizing classifier performance via an approximation to the Wilcoxon-Mann-Whitney Statistic.

[58] Ashburner J, Klöppel S (2011). Multivariate models of inter-subject anatomical variability. *NeuroImage*.

[59] Holmes AP, Blair RC, Watson JDG, Ford I (1996). Nonparametric analysis of statistic images from functional mapping experiments. *Journal of Cerebral Blood Flow and Metabolism*.

[60] Nichols TE, Holmes AP (2002). Nonparametric permutation tests for functional neuroimaging: a primer with examples. *Human Brain Mapping*.

[61] Eklund A, Andersson M, Knutsson H (2011). Fast random permutation tests enable objective evaluation of methods for single subject fMRI analysis. *International Journal of Biomedical Imaging*.

[62] Golland P, Fischl B Permutation tests for classification: towards statistical significance in image-based studies.

[63] Golland P, Liang F, Mukherjee S, Panchenko D Permutation tests for classification.

[64] Smith AM, Lewis BK, Ruttimann UE (1999). Investigation of low frequency drift in fMRI signal. *NeuroImage*.

